# Angular‐Momentum Transfer Mediated by a Vibronic‐Bound‐State

**DOI:** 10.1002/advs.202304698

**Published:** 2023-11-09

**Authors:** Yun‐Yi Pai, Claire E. Marvinney, Ganesh Pokharel, Jie Xing, Haoxiang Li, Xun Li, Michael Chilcote, Matthew Brahlek, Lucas Lindsay, Hu Miao, Athena S. Sefat, David Parker, Stephen D. Wilson, Jason S. Gardner, Liangbo Liang, Benjamin J. Lawrie

**Affiliations:** ^1^ Materials Science and Technology Division Oak Ridge National Laboratory Oak Ridge TN 37831 USA; ^2^ Materials Department University of California Santa Barbara Santa Barbara CA 93106 USA; ^3^ Center for Nanophase Materials Sciences Oak Ridge National Laboratory Oak Ridge TN 37831 USA

**Keywords:** phonon circularity, quantum spin liquid, Raman microscopy, vibronic bound state

## Abstract

The notion that phonons can carry pseudo‐angular momentum has many major consequences, including topologically protected phonon chirality, Berry curvature of phonon band structure, and the phonon Hall effect. When a phonon is resonantly coupled to an orbital state split by its crystal field environment, a so‐called vibronic bound state forms. Here, a vibronic bound state is observed in NaYbSe_2_, a quantum spin liquid candidate. In addition, field and polarization dependent Raman microscopy is used to probe an angular momentum transfer of Δ**J**_*z*_ = ±ℏ between phonons and the crystalline electric field mediated by the vibronic bound stat. This angular momentum transfer between electronic and lattice subsystems provides new pathways for selective optical addressability of phononic angular momentum via electronic ancillary states.

## Graphical abstract

Angular momentum transfer between phonons and the crystal electric field in a candidate quantum spin liquid is observed with polarization‐ and magnetic‐field‐dependent Raman microscopy. These results provide a new framework for selective optical addressability of phononic angular momentum via electronic, spin, and orbital degrees of freedom in emerging quantum materials.

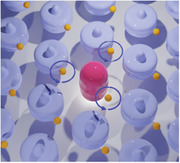

## 1 Introduction

Since the seminal work of Bloch in 1929,^[^ ^1^ ^]^ electron–phonon coupling has emerged as one of the most important topics in modern condensed matter physics. In conventional superconductors, electron‐phonon coupling underlies Cooper pairing. In semiconductors, it sets an upper bound for electron mobility. Electron–phonon coupling also governs numerous thermal and spin relaxation processes in solids. In ionic crystals with f orbitals, spin‐orbit coupling and the crystal electric field (CEF) associated with the ionic or ligand environment split the electronic wavefunction eigenstates into manifolds. Atomic displacements can change the ligand environment and hence the orbital manifolds. This change is typically treated within the adiabatic Born–Oppenheimer approximation, in which atomic motion is slow relative to the electronic degrees of freedom. However, when phonon‐CEF coupling is nearly resonant, the Born–Oppenheimer approximation is inadequate and a new state—a vibronic bound state (VBS), with both orbital character and phonon character—can form.

**Figure 1:**
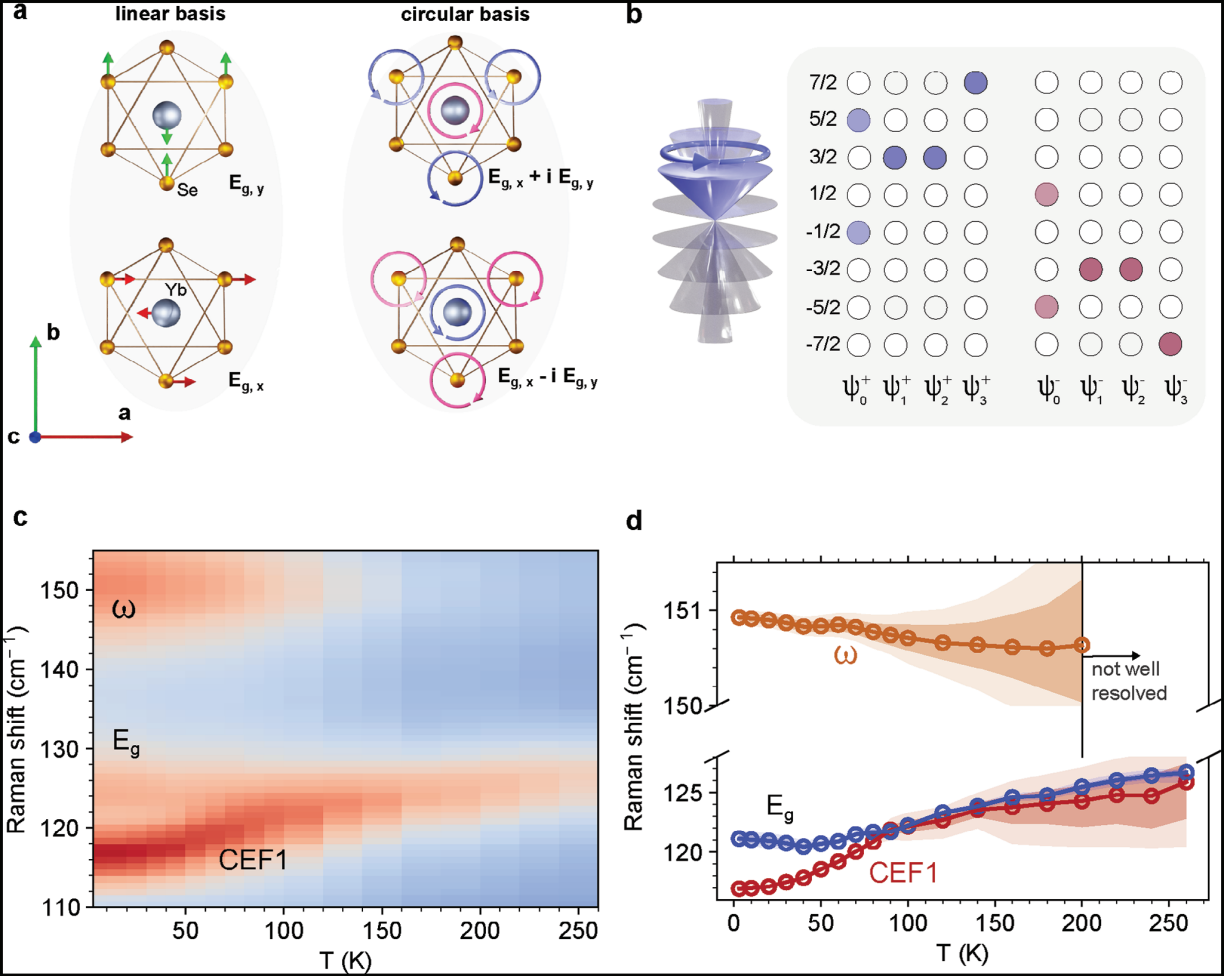
a) The *E*_g_ eigenspace is spanned by the basis {$E$_g, $x$_, $E$_g, $y$_}, which can also be spanned by {$E$_g, +_, $E$_g, $‐$_} = {$E$_g, $x$_ + *i*$E$_g, $y$_, $E$_g, $x$_ − *i*$E$_g, $y$_}. b) Representations of the CEF eigenstates $|\psi_{0}^{\pm }\rangle$, $|\psi_{1}^{\pm }\rangle$, $|\psi_{2}^{\pm }\rangle$, and $|\psi_{3}^{\pm }\rangle$. The eigenstates are linear combinations of multiplets *m*_*J*_ = −7/2…7/2. For example, $|\psi_{1}^{+}\rangle =|\frac{7}{2},\frac{3}{2}\rangle$ is represented by precessing cones with $c_{\frac{3}{2}}\neq 0$ for $|\psi_{1}^{+}\rangle =c_{\frac{3}{2}}\left|J,\frac{3}{2}\right\rangle$. Hence, all but the $m_{J}=\frac{3}{2}$ cones are transparent (and all but the $m_{J}=\frac{3}{2}$ circles are empty). The same follows for $|\psi_{1}^{-}\rangle$ and $|\psi_{0,2,3}^{\pm }\rangle$. c) Temperature dependent Raman spectra for CEF1, $E$_g_, and the VBS, ω, from *T* = 3.3 K to *T* = 260 K. d) Peak positions for CEF1, *E*_g_ and ω extracted from Bayesian inference. The symbols are medians of the posterior distribution, and the 68% and 95% HDIs are represented by darker and lighter shading, respectively.

Phonons can carry pseudo‐angular momentum when the 2D eigenvibration can be represented by a circular basis, as shown in **Figure** **1**a.^[^ ^2^, ^3^, ^4^, ^5^, ^6^, ^7^, ^8^, ^9^ ^]^ The conceptual difference between angular momentum and pseudo angular momentum is similar to that between linear momentum and pseudo linear momentum. The prefix *pseudo* is unnecessary for elementary particles in a vacuum and distinguishes that from motion in a medium, such as a lattice. Regular angular momentum exhibits rotational invariance under the rotation of the entire system while *pseudo* angular momentum is invariant under the rotation of the field.^[^ ^10^ ^]^

The ability to control angular‐momentum transfer between phonons and electronic degrees of freedom may unlock new opportunities in quantum information processing by providing new interfaces with spins in materials. However, the transfer of angular momentum between photons, electronic spin, orbital excitations, and phonons is still not well understood. It has been studied, for instance, in the context of paramagnetic spin relaxation,^[^ ^11^, ^12^ ^]^ ultra‐fast demagnetization processes,^[^ ^13^, ^14^ ^]^ and recently *angulon* quasiparticles.^[^ ^15^ ^]^ Phononic angular momentum is key to several fundamental effects in physics, including the microscopic explanation of the phonon Hall effect,^[^ ^16^ ^]^ the Einstein‐de Haas effect,^[^ ^17^, ^18^, ^19^ ^]^ and zero‐point energies of chiral phonons.^[^ ^17^ ^]^

NaYbSe_2_^[^ ^20^, ^21^, ^22^, ^23^, ^24^ ^]^ belongs to the family of the form A^1 +^Yb^3 +^X${}_{2}^{2-}$ that have been identified as quantum spin liquid (QSL) candidates. 14 members of this family have been identified to date.^[^ ^21^, ^22^, ^23^, ^25^, ^26^, ^27^, ^28^, ^29^, ^30^, ^31^, ^32^, ^33^, ^34^, ^35^, ^36^, ^37^ ^]^ They all have planar triangular lattices decorated by antiferromagnetically coupled spins making them susceptible to geometric frustration, resulting in a lack of long‐range magnetic order down to the lowest probed temperatures. This family of candidate QSLs is objectively less defect prone than Yb(Mg, Ga)O_4_,^[^ ^23^ ^]^ and the breadth of substitutional composites in the family makes it an ideal platform for the study and control of QSL excitations.

The effective spin S_eff_ = 1/2 of the system comes from the Yb^3 +^ ion, which has the [Xe]4f^13^ electronic configuration. Yb^3 +^, like all the elements in the 4f block, has weak exchange coupling and strong spin‐orbit coupling compared to the 3d‐block elements. The ground state spin‐orbit manifold of Yb^3 +^ has total spin *J* = 7/2. The next manifold *J* = 5/2 is more than an eV above the ground state manifold.^[^ ^38^, ^39^ ^]^ The ground state spin‐orbit manifold *J* = 7/2 splits into four Kramers pairs $|\psi_{0}^{\pm }\rangle$, $|\psi_{1}^{\pm }\rangle$, $|\psi_{2}^{\pm }\rangle$, $|\psi_{3}^{\pm }\rangle$, where the *z*‐component of the total angular momentum in the system can take values of m_J_ = ±1/2, ±3/2, ±5/2, and ±7/2. CEF1, CEF2, and CEF3 describe the transition between the excited doublets and the ground state $|\psi_{0}^{\pm }\rangle$, respectively. The CEF splitting within the ground‐state manifold is typically only tens of meV due to the well‐shielded nature of the 4f electrons. Structurally, NaYbSe_2_ has less distortion in its YbSe_6_ octahedra than other members in the A^1 +^Yb^3 +^X${}_{2}^{2-}$ family.^[^ ^25^ ^]^ Its ground states are reported to be described by spinon excitations^[^ ^21^ ^]^ or ferrimagnetic quasistatic and dynamic excitations within a QSL matrix.^[^ ^40^ ^]^ While pristine NaYbSe_2_ is insulating, with increasing pressure it exhibits increased conductivity and eventually demonstrates a dip in resistivity attributed to a possible superconducting state.^[^ ^20^, ^24^ ^]^

Elementary excitations in solids like magnons, phonons, and CEFs are usually considered decoupled and determined independently. However, strong coupling between normally decoupled excitations can result in fundamentally new material functionality. Thalmeier and Fulde^[^ ^41^ ^]^ first theoretically described how a bound state between a crystal field excitation and phonons may form, but VBSs have only been reported in a handful of intermetallics^[^ ^41^, ^42^, ^43^, ^44^, ^45^, ^46^, ^47^ ^]^ and oxides,^[^ ^48^, ^49^, ^50^ ^]^ including Tb_2_Ti_2_O_7_^[^ ^51^ ^]^ another geometrically frustrated magnet whose quantum spin liquid state is not yet fully understood.^[^ ^52^, ^53^ ^]^ Here we report the presence of a VBS in NaYbSe_2_ and a change of angular momentum Δ**J**_*z*_ = ±1ℏ in an orbital excitation due to phononic coupling.

## 2 Results and Discussion

We first verify the primary CEF excitations and phonon modes with temperature‐dependent Raman spectroscopy. The temperature dependence of the relevant Raman features is depicted in Figure 1c while the full spectra are shown in Figures S2 and S3, Supporting Information. Consistent with earlier neutron scattering and Raman reports,^[^ ^23^, ^35^ ^]^ strong CEF modes are observed at 117.2 cm^−1^ (CEF1), 197.8 cm^−1^ (CEF2), and 247.0 cm^−1^ (CEF3). These become significantly stronger in intensity and soften (shift toward lower energy) as the temperature decreases. NaYbSe_2_ also has two Raman‐active phonon modes: *E*_g_ at 124.4 cm^−1^ and *A*_1g_ at 172.8 cm^−1^ within this frequency space. The intensity and energy of these phonon modes exhibit a substantially weaker temperature dependence. Figure 1b shows a classical *spinning top* representation of the spin‐orbit eigenstates for $|\psi_{0}^{+}\rangle$ as well as the relative weights $c_{m_{j}}$ for $|\psi_{0}^{\pm }\rangle$, $|\psi_{1}^{\pm }\rangle$, $|\psi_{2}^{\pm }\rangle$, $|\psi_{3}^{\pm }\rangle$ as |ψ〉= $\sum_{m_{j}=-\frac{7}{2},\text{…}\frac{7}{2}}c_{m_{j}}\left|\frac{7}{2},m_{j}\right\rangle$. Empty (filled) circles represent $c_{m_{j}}=0$ ($c_{m_{j}}\neq 0$). The blue (pink) cones represent the + (−) branch. Note that the *E*_g_ and CEF1 modes exist at similar energies. This is the case for NaYbSe_2_,^[^ ^23^ ^]^ CsYbSe_2_,^[^ ^35^ ^]^ and KYbSe_2_.^[^ ^37^ ^]^ Using Bayesian inference with a Hamiltonian Monte Carlo model in PyMC3,^[^ ^54^ ^]^ we track the peak parameters from the experimental data shown in Figure 1c. The extracted peak positions for CEF1 and *E*_g_ are shown in Figure 1d. The symbols represent the median values from the Bayesian inference. The darker shaded bands illustrate the 68% (corresponding to 1σ in the central limit) highest density intervals (HDIs), and the lighter shaded bands illustrate the 95% (corresponding to 2σ) HDIs. Other selected modes are described in the Supporting Information. The model has larger error bars from *T* = 175 to 255 K, but the modes are well resolved elsewhere.

A previously unreported mode is clearly seen in our data at 151.2 cm^−1^. Labeled ω in Figure 1c,d, this mode has characteristics in common with both CEFs and phonons: ω has a significantly stronger structure factor at low temperatures, just as the CEFs do, but it does not exhibit the strong softening at lower temperatures that the CEF modes exhibit. Instead, it hardens in a manner consistent with slight phonon hardening in NaYbSe_2_ at low temperatures. We therefore assign ω as a VBS with parent states CEF1 and *E*_g_. Earlier Raman spectra in this temperature range^[^ ^23^ ^]^ were only helicity‐resolved at room temperature, where ω is weak.

In order to better understand the origin of the ω mode, we calculated the phonon dispersion relationship for NaYbSe_2_ using density functional theory (DFT) and Perdew–Burke–Ernzerhof (PBE) exchange energy, as shown in Figure S1, Supporting Information. These calculations suggest that no optical or acoustic phonons are present near ω. If anything, PBE normally overestimates the in‐plane lattice constants, resulting in softer in‐plane vibrations (*E*_g_) than those measured. Comparing these calculations to published data (and the data in this manuscript) the calculated *E*_g_ at the gamma point is indeed 15 cm^−1^ softer than that measured. The *A*_1g_ mode is also calculated to be softer than the measured mode, 135 cm^−1^ compared to the measured 172.8 cm^−1^. Between these two energies, there are very few modes at the gamma point, and halfway between them there are no phonon branches.

**Figure 2:**
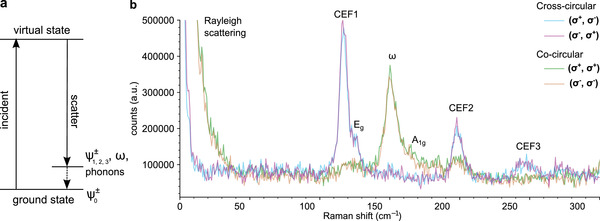
a) Schematic illustration of Raman scattering where CEF1‐3, phonon modes, and ω can be excited in the process. b) Helicity‐resolved Raman spectra at *T* = 4 K, *B* = 0 T. Four prominent modes are CEF1 (with an *E*_g_ phonon mode at its shoulder), ω, CEF2, and CEF3. The Rayleigh scattering and ω are of (σ^+^, σ^+^) and (σ^−^, σ^−^) co‐circular scattering channels, while CEF1‐3 and *E*_g_ are of (σ^+^, σ^−^) and (σ^−^, σ^+^) cross‐circular channels.

In fact, most models have no phonons throughout the Brillouin zone around 150 cm^−1^. Additionally, published neutron scattering data suggests there are no phonons in this frequency space. Specifically, figure S3J in ref. [21] reveals a decrease in spectral weight as the temperature increases. Additionally, Zhang et al.^[^ ^23^ ^]^ could not model extra spectral weight at ≈19 meV (see figure 2 of that manuscript) when the temperature decreased below 100 K. Notably, these neutron data have momentum transfer dependence like a phonon, but the temperature dependence of a crystal field, consistent with a vibronic bound state.

The mode ω is present in both NaYbSe_2_ and CsYbSe_2_, although it is far less prominent in CsYbSe_2_.^[^ ^35^ ^]^ This is likely because in NaYbSe_2_, Na is both smaller and lighter. The lattice is therefore more tightly‐packed. The lighter Na also has larger vibrational displacements. Both effects increase the coupling strength, resulting in a VBS far stronger than that in CsYbSe_2_. Fitting to the Thalmeier–Fulde description of a magnetoelastic vibronic bound state^[^ ^41^ ^]^ yields a coupling strength of 32.0 cm^−1^ (3.97 meV) for NaYbSe_2_, which is stronger than the 23.6 cm^−1^ (2.93 meV) coupling strength reported for CsYbSe_2_ and comparable to the roughly 34.0 cm^−1^ (4.22 meV) coupling strength reported for Ce_2_O_3_.^[^ ^43^ ^]^

To gain a deeper understanding of the CEF and the ω Raman modes, we studied the helicity dependence and magnetic field dependence of Raman spectra of NaYbSe_2_. During the Raman scattering process, the energy transferred from the photons can be used to excite a variety of (quasi)particles in the system, including electronic orbitals, phonons, and their hybridized states, as indicated by the schematic illustration in **Figure** **2**a. Figure 2b shows helicity‐resolved Raman spectra acquired at *T* = 4 K, with CEF1, CEF2, CEF3, *E*_g_, *A*_1g_, and ω highlighted. Here, we can simultaneously observe electronic orbital excitations (CEF1, CEF2, and CEF3), phononic excitations (*E*_g_ and *A*_1g_), and the entangled state ω between the orbital and phononic excitations by low‐temperature Raman spectroscopy. Furthermore, we discovered that these (quasi)particles exhibit different responses to the helicity of photons. While *A*_1g_ and ω (along with the Rayleigh scattering; see Figure S7, Supporting Information) are stronger in the co‐circular polarization configuration, CEF1–CEF3 and *E*_g_ are stronger in the cross‐circular polarization configuration.

**Figure 3:**
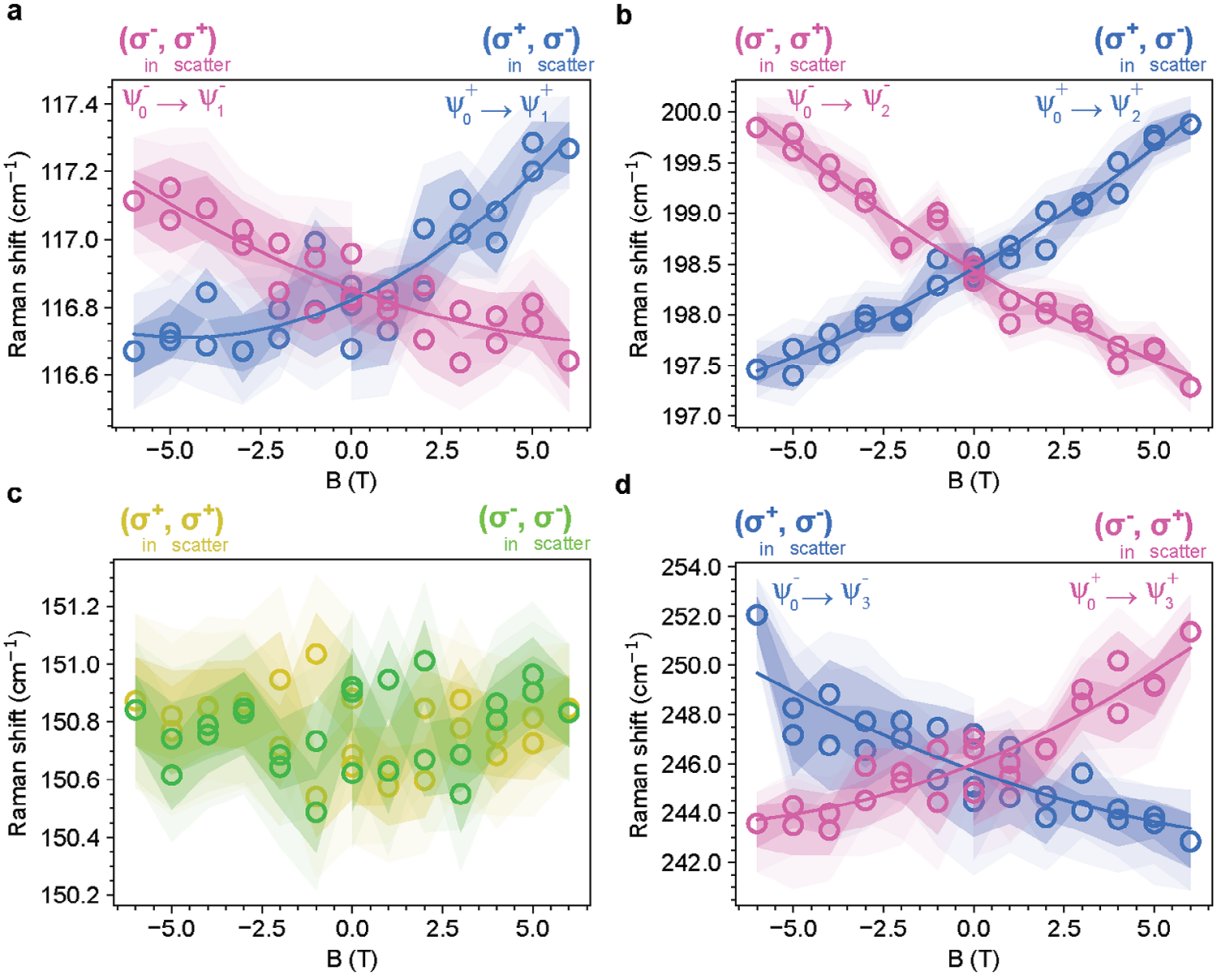
Helicity‐resolved magnetic field dependence of CEFs and ω for NaYbSe_2_ at *T* = 4 K. a) CEF1, b) CEF2, c) ω, and d) CEF3. The 68% and 95% HDIs are represented by darker and lighter shading, respectively. See Supporting Information for the raw spectra that the peak positions were extracted from.

We also performed magnetic field‐dependent Raman spectroscopy with **B** || **c**, which lifts the degeneracy within each of the Kramers pairs and therefore provides further information on the orbital excitations corresponding to CEF1–3. Magnetization and specific heat measurements have been previously used to show that no field‐induced ordering is present in NaYbSe_2_ for **B** || **c** for *B* < 9 T at *T* = 4 K.^[^ ^22^ ^]^ Therefore, with the 6 T magnetic fields accessible here, no field‐induced transition is expected. **Figure** **3** shows the magnetic field dependence of the CEF levels for CEF1 (Figure 3a), CEF2 (Figure 3b), ω (Figure 3c), and CEF3 (Figure 3d) measurements. Again, the CEF levels only show up in cross‐circular polarization configurations, (σ^+^, σ^−^) and (σ^−^, σ^+^), while the ω mode only appears in co‐circular polarization configurations, (σ^+^, σ^+^) and (σ^−^, σ^−^). Note that in the (σ^+^, σ^−^) configuration and with increasing positive magnetic field, CEF1 and CEF2 increase in energy while CEF3 decreases. This dependence is inverted if the magnetic field is reversed (*B* → −*B*) or the helicity is reversed ((σ^+^, σ^−^) → (σ^−^, σ^+^)).

The observed circular polarization dependence and magnetic field dependence of CEF1–3 and ω in Figures 2 and 3 clearly indicate different underlying optical selection rules that can be understood based on angular momentum conservation. Each left (right) circularly polarized σ^+^ (σ^−^) photon carries angular momentum +ℏ (−ℏ).^[^ ^55^ ^]^ The link between helicity and circular polarization is given by *h* = **σ** · **k**, where **k** is the momentum of the photon, and *h* the helicity.^[^ ^56^ ^]^ Absorption of a σ^+^ photon increases the angular momentum of the system by +ℏ, corresponding to being acted on by operator *J*_+_, while scattering a σ^+^ photon corresponds to *J*_−_ acting on the system. Therefore, if one considers Raman spectra acquired in the (σ_incident_, σ_scatter_) = (σ^+^, σ^−^) configuration where the change of angular momentum in photons is Δ*J*_photon_ = −2ℏ, the system could obtain +2ℏ of angular momentum from the photons (i.e., Δ*J*_system_ = +2ℏ), corresponding to *J*_+_ *J*_+_ acting on the system (a generalization of the closure condition in Axe et al.,^[^ ^57^ ^]^ which contracts ∑_virtual_||〈ψ_final_|*M*_*i*_|ψ_virtual_〉〈ψ_virtual_|*M*_*j*_|ψ_initial_〉|| to ||〈ψ_final_|*M*_*i*_ *M*_*j*_|ψ_initial_〉|| for any dipolar transition *M*_*i*_, *M*_*j*_). Therefore, a mode that is active in the (σ^+^, σ^−^) configuration could have dominant matrix‐element contributions from ||〈ψ_final_|*J*_+_ *J*_+_|ψ_initial_〉||. However, it is important to note that in an analog to the Umklapp process, the threefold rotational symmetry in NaYbSe_2_^[^ ^58^ ^]^ allows for discrete angular momentum conservation as long as |Δ*J*_photon_ + Δ*J*_system_|/ℏ = 0 (modulo 3). In other words, the angular momentum conservation rule is relaxed so that the total change of angular momentum can be either zero or a multiple of 3ℏ due to the threefold rotational symmetry.^[^ ^59^, ^60^, ^61^ ^]^ This means that in the (σ^+^, σ^−^) configuration where Δ*J*_photon_ = −2ℏ, Δ*J*_system_ can be +2ℏ or −ℏ to satisfy the relaxed angular momentum conservation rule. For Δ*J*_system_ = +2ℏ, it corresponds to the operation of *J*_+_ *J*_+_, while for Δ*J*_system_ = −ℏ, it corresponds to *J*_−_. Therefore, a mode that is active in the (σ^+^, σ^−^) configuration could also have dominant matrix‐element contributions from ||〈ψ_final_|*J*_−_|ψ_initial_〉||. In contrast, in the (σ_incident_, (σ^−^, σ^+^) configuration where Δ*J*_photon_ = +2ℏ, the system could lose 2ℏ of angular momentum to the photons (Δ*J*_system_ = −2ℏ), corresponding to *J*_−_ *J*_−_ acting on the system, or Δ*J*_system_ = +ℏ, corresponding to *J*_+_ acting on the system. Hence, a mode that is active in the (σ^−^, σ^+^) configuration should have dominant matrix‐element contributions from ||〈ψ_final_|*J*_−_ *J*_−_|ψ_initial_〉|| or ||〈ψ_final_|*J*_+_|ψ_initial_〉||.

Since all the CEF modes are enhanced in the cross‐circular channel and suppressed in the co‐circular channel, all of them should have a finite change of angular momentum after the orbital transitions. As discussed above, Δ*J*_CEF_ = +2ℏ or −ℏ in the (σ^+^, σ^−^) configuration, while Δ*J*_CEF_ = −2ℏ or +ℏ in the (σ^−^, σ^+^) configuration. Considering that an additional Zeeman dependence *H*_**B**_ = −*g*_*J*_μ_B_ **J**_*z*_ · **B** is added when a magnetic field is applied, the change of the sign of Δ*J*_CEF_ from (σ^+^, σ^−^) to (σ^−^, σ^+^) explains why the magnetic field dependence of CEF1–3 is inverted when the helicity is reversed in Figure 3. Another intriguing finding from Figure 3 is that the magnetic field dependence of CEF1 and CEF2 is opposite to that of CEF3 regardless of the helicity, suggesting that Δ*J*_CEF1_ and Δ*J*_CEF2_ share the same sign, whereas Δ*J*_CEF3_ has the opposite sign. More specifically, in the (σ^+^, σ^−^) configuration, we should have Δ*J*_CEF1_ = Δ*J*_CEF2_ = +2ℏ while Δ*J*_CEF3_ = −ℏ, or Δ*J*_CEF1_ = Δ*J*_CEF2_ = −ℏ while Δ*J*_CEF3_ = +2ℏ. Note that Δ*J* = −ℏ is allowed for a CEF level due to the threefold rotational symmetry that relaxes the angular momentum conservation rule, but we expect that Δ*J* = +2ℏ that satisfies the strict angular momentum conservation should have a higher probability and stronger Raman signals. Since CEF1 and CEF2 have much stronger Raman intensities than CEF3 (Figure 2b), it is natural to conclude that Δ*J*_CEF1_ = Δ*J*_CEF2_ = +2ℏ while Δ*J*_CEF3_ = −ℏ in the (σ^+^, σ^−^) configuration. Similarly, Δ*J*_CEF1_ = Δ*J*_CEF2_ = −2ℏ while Δ*J*_CEF3_ = +ℏ in the (σ^−^, σ^+^) configuration. In terms of matrix operations, $\left\langle \psi_{1}^{\pm }|J_{+}J_{+}|\psi_{0}^{\pm }\right\rangle$, $\left\langle \psi_{1}^{\pm }|J_{-}J_{-}|\psi_{0}^{\pm }\right\rangle$, $\left\langle \psi_{2}^{\pm }|J_{+}J_{+}|\psi_{0}^{\pm }\right\rangle$, $\left\langle \psi_{2}^{\pm }|J_{-}J_{-}|\psi_{0}^{\pm }\right\rangle$, $\left\langle \psi_{3}^{\pm }|J_{-}|\psi_{0}^{\pm }\right\rangle$, and $\left\langle \psi_{3}^{\pm }|J_{+}|\psi_{0}^{\pm }\right\rangle$ should be significant while $\left\langle \psi_{3}^{\pm }|J_{+}J_{+}|\psi_{0}^{\pm }\right\rangle$, $\left\langle \psi_{3}^{\pm }|J_{-}J_{-}|\psi_{0}^{\pm }\right\rangle$, $\left\langle \psi_{1}^{\pm }|J_{+}J_{-}|\psi_{0}^{\pm }\right\rangle$, $\left\langle \psi_{1}^{\pm }|J_{-}J_{+}|\psi_{0}^{\pm }\right\rangle$, $\left\langle \psi_{2}^{\pm }|J_{+}J_{-}|\psi_{0}^{\pm }\right\rangle$, $\left\langle \psi_{2}^{\pm }|J_{-}J_{+}|\psi_{0}^{\pm }\right\rangle$, $\left\langle \psi_{3}^{\pm }|J_{+}J_{-}|\psi_{0}^{\pm }\right\rangle$, and $\left\langle \psi_{3}^{\pm }|J_{-}J_{+}|\psi_{0}^{\pm }\right\rangle$ should all be small or zero (the last six matrix elements correspond to the co‐circular polarization).

**Figure 4:**
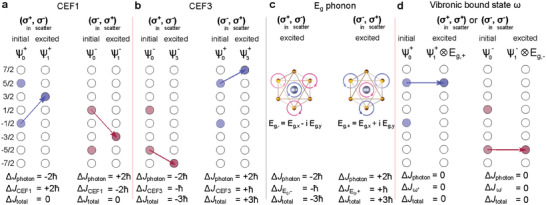
a) Selection rule for CEF1 corresponding to $|\psi_{0}^{+}\rangle \to |\psi_{1}^{+}\rangle$ or $|\psi_{0}^{-}\rangle \to |\psi_{1}^{-}\rangle$. A similar rule applies for CEF2, although the transition corresponds to $|\psi_{0}^{+}\rangle \to |\psi_{2}^{+}\rangle$ or $|\psi_{0}^{-}\rangle \to |\psi_{2}^{-}\rangle$. b) Selection rule for CEF3 corresponding to $|\psi_{0}^{-}\rangle \to |\psi_{3}^{-}\rangle$ or $|\psi_{0}^{+}\rangle \to |\psi_{3}^{+}\rangle$. Note that the threefold rotational symmetry in NaYbSe_2_ allows for discrete angular momentum conservation: |Δ*J*_photon_ + Δ*J*_particle_|/ℏ = 0 (modulo 3). Here Δ*J*_total_ = −3ℏ or Δ*J*_total_ = 3ℏ. c) Selection rule for the doubly degenerate *E*_g_ mode that can have (pseudo)angular momentum of ±ℏ. d) Selection rule for the vibronic bound state ω. Due to the angular momentum transfer between the CEF1 and *E*_g_ mode, the excited state of ω, $|\psi_{1}^{+}\rangle ⊗|E_{\text{g,+}}\rangle$ or $|\psi_{1}^{-}\rangle ⊗|E_{\text{g,}-}\rangle$, has a completely different helicity selection rule and magnetic field dependence than CEF1.

As shown in Figure 1b, the eigenstates of CEF levels are linear combinations of multiplets *m*_*J*_ = −7/2…7/2: |ψ^±^〉 = $\sum_{m_{j}=-\frac{7}{2},\text{…}\frac{7}{2}}c_{m_{j}}^{\pm }\left|\frac{7}{2},m_{j}\right\rangle$. Typically, CEF parameters are fit to a CEF Hamiltonian with Stevens operators given by the symmetry of the ionic environment of the 4f orbitals, as described in the Experimental Section. The procedure is frequently under‐constrained, and there are enough degrees of freedom to fit experimentally observed CEF energy levels with more than one set of CEF parameters that minimize the error that may exist. The order of the eigenstates may not be the same across those sets. However, once the constraints imposed by the optical selection rules discussed above are taken into account, we are able to find a set of CEF parameters, as indicated by filled and empty circles in Figure 1b, that can explain the observed helicity and magnetic field dependence in Figures 2 and 3, and we can make final assignments of CEF1‐3. As illustrated in **Figure** **4**a, CEF1 comes from $|\psi_{0}^{+}\rangle \to |\psi_{1}^{+}\rangle$ in the (σ^+^, σ^−^) configuration, where Δ*J*_photon_ = −2ℏ and Δ*J*_CEF1_ = +2ℏ, or $|\psi_{0}^{-}\rangle \to |\psi_{1}^{-}\rangle$ in the (σ^−^, σ^+^) configuration, where Δ*J*_photon_ = +2ℏ and Δ*J*_CEF1_ = −2ℏ (see the arrows for the transitions). We note that the transition of $|\psi_{0}^{+}\rangle \to |\psi_{1}^{-}\rangle$ with Δ*J*_CEF1_ = −ℏ is allowed in (σ^+^, σ^−^) and the transition of $|\psi_{0}^{-}\rangle \to |\psi_{1}^{+}\rangle$ with Δ*J*_CEF1_ = +ℏ is allowed in (σ^−^, σ^+^), since Δ*J*_total_ = −3ℏ and +3ℏ, respectively, which satisfies the discrete angular momentum conservation. However, these transitions allowed by the crystal threefold rotational symmetry are of a higher order and should be much weaker. Similar transitions apply for CEF2, although they correspond to $|\psi_{0}^{+}\rangle \to |\psi_{2}^{+}\rangle$ or $|\psi_{0}^{-}\rangle \to |\psi_{2}^{-}\rangle$. For CEF3 in Figure 4b, however, any transition with Δ*J* = −2ℏ or +2ℏ is impossible; hence, the transition of $|\psi_{0}^{-}\rangle \to |\psi_{3}^{-}\rangle$ with Δ*J*_CEF3_ = −ℏ is the only one allowed in (σ^+^, σ^−^) where Δ*J*_total_ = −3ℏ, and the transition of $|\psi_{0}^{+}\rangle \to |\psi_{3}^{+}\rangle$ with Δ*J*_CEF3_ = +ℏ is the only one allowed in (σ^−^, σ^+^) where Δ*J*_total_ = +3ℏ. This could explain why CEF3 is much weaker than CEF1 and CEF2.

As for the phonon Raman modes, in general, a nondegenerate Γ‐point phonon (like the *A*_1g_ Raman mode in NaYbSe_2_) cannot have angular momentum since the eigenvector is a real number. It is therefore only observed in the co‐circular polarization configuration, as shown in Figure 2b. However, when the Γ‐point phonon is doubly degenerate, the two real eigenvectors can be reconstructed by a complex superposition as shown in Figure 1a. Therefore, doubly degenerate modes like the *E*_g_ Raman mode in NaYbSe_2_ can have (pseudo)angular momentum of ±ℏ.^[^ ^3^, ^9^, ^60^, ^62^ ^]^ With nonzero angular momentum, it can change the chirality of incident photons and appear in the cross‐circular polarization configuration. As shown in Figure 4c, in the (σ^+^, σ^−^) configuration, Δ*J*_photon_ = −2ℏ and $\Delta J_{E_{\text{g,}-}}=-\hbar$, so it is allowed by the discrete angular momentum conservation as Δ*J*_total_ = −3ℏ; In the (σ^−^, σ^+^) configuration, Δ*J*_photon_ = +2ℏ and $\Delta J_{E_{\text{g,+}}}=+\hbar$, giving rise to Δ*J*_total_ = +3ℏ. Such an optical selection rule is similar to that shown for CEF3 in Figure 4b, which explains why both are observed in the cross‐circular polarization. Similar results have been reported for *E* symmetry Raman modes in TMDs, CrBr_3_, and quartz, among other materials.^[^ ^9^, ^60^, ^61^ ^]^

Finally, moving on to the VBS ω, it is much stronger in the co‐circular polarization configuration and its magnetic field dependence is weak (Figures 2 and 3), in stark contrast to CEF1‐3. This indicates that the change in angular momentum for ω is zero: Δ**J**_*z*_ = 0. Since it is a coupled state between the CEF1 and the *E*_g_ mode, we consider the 2 × ~2 dimensional space $|\psi_{1}^{\pm }\rangle ⊗|E_{\text{g,}\pm }\rangle$. As the angular momentum change Δ**J**_*z*_ for CEF1 is finite (Figure 4a), the phonon subsystem has to carry additional angular momentum (Figure 4c) in order to yield zero angular momentum for ω. Therefore, the circular basis {$E$_g, +_, $E$_g, $‐$_} is the natural choice for describing the eigenvibration. As discussed previously, CEF1 can arise from four possible transitions while *E*_g_ can be $E$_g, +_ and $E$_g, $‐$_, which gives rise to a total of eight possible transitions for ω. Based on the angular momentum conservation rule, however, we found that two states, $\omega^{+}=|\psi_{0}^{+}\rangle \to |\psi_{1}^{+}\rangle ⊗|E_{\text{g,+}}\rangle$ and $\omega^{-}=|\psi_{0}^{-}\rangle \to |\psi_{1}^{-}\rangle ⊗|E_{\text{g,}-}\rangle$, are the most probable (see more details in the Supporting Information). As shown in Figure 4d, due to the angular momentum transfer between the CEF1 and *E*_g_ mode, the excited state of ω, $|\psi_{1}^{+}\rangle ⊗|E_{\text{g,+}}\rangle$ or $|\psi_{1}^{-}\rangle ⊗|E_{\text{g,}-}\rangle$, has the *z*‐component of the angular momentum effectively raised or lowered by ℏ compared to the $|\psi_{1}^{+}\rangle$ or $|\psi_{1}^{-}\rangle$ shown in Figure 4a, respectively. As a result, the transitions of $|\psi_{0}^{+}\rangle \to |\psi_{1}^{+}\rangle ⊗|E_{\text{g,+}}\rangle$ and $|\psi_{0}^{-}\rangle \to |\psi_{1}^{-}\rangle ⊗|E_{\text{g,}-}\rangle$ have Δ**J**_*z*_ = 0 to satisfy the selection rule in the co‐circular polarization configuration and explain the weak magnetic field dependence.

**Figure 5:**
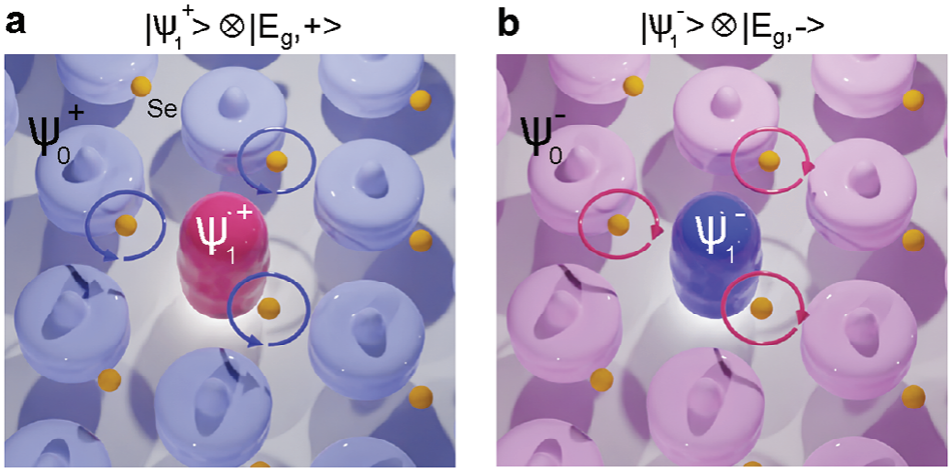
Schematic illustrations of the vibronic bound state ω: a) $|\psi_{0}^{+}\rangle \to |\psi_{1}^{+}\rangle ⊗|E_{\text{g,+}}\rangle$ and b) $|\psi_{0}^{-}\rangle \to |\psi_{1}^{-}\rangle ⊗|E_{\text{g,}-}\rangle$.

**Figure** **5**a,b illustrates the two states, $|\psi_{1}^{+}\rangle ⊗|E_{\text{g,+}}\rangle$ and $|\psi_{1}^{-}\rangle ⊗|E_{\text{g,}-}\rangle$ based on the observations described above. In Figure 5a, when the ground state is $|\psi_{0}^{+}\rangle$ (shown in light pink), the *J*_+_ *J*_−_ or *J*_−_ *J*_+_ operator excites the system to the + branch, $|\psi_{1}^{+}\rangle ⊗|E_{\text{g,+}}\rangle$ (where the CEF excitation corresponds to the + branch and the phononic excitation corresponds to + angular momentum). Figure 5b shows the same effect for $|\psi_{0}^{-}\rangle$ to $|\psi_{1}^{-}\rangle ⊗|E_{\text{g,}-}\rangle$. Because the transition is degenerate for $|\psi_{0}^{\mp }\rangle \to \omega^{\pm }$ with *J*_+_ *J*_−_ or *J*_−_ *J*_+_, this eigenspace, in principle, can be transduced from a QSL ground state. If the QSL ground state is written as $\sum_{j}\prod_{i}\psi_{0}^{\sigma_{ij}}$ – where *i* is the site index, *j* the configuration index, and σ_*ij*_ = +, − is the branch—then *J*_+_ *J*_−_ or *J*_−_ *J*_+_ should bring the system into $\sum_{j}\prod_{i}\omega^{\sigma_{ij}}$ within the point spread volume of the excitation beam, transducing the possible ground state entanglement to the VBS ω^±^.

It is worth noting that, in the original Thalmeier–Fulde model, two VBS modes are expected to emerge from the hybridization of a CEF mode and a phonon mode.^[^ ^41^ ^]^ Besides the two most probable VBS transitions shown in Figure 4d that give rise to the observed ω peak in the Raman spectra, there are six other transitions that could contribute to the other VBS mode that is not observed in our Raman measurements (see Equation (S1), Supporting Information). Since the picture of how orbital and phononic excitations couple is not entirely clear, a definitive answer to why other transitions and the other VBS mode do not appear in Raman scattering is beyond the scope of this work. It is expected that the electron–phonon coupling may be more complicated compared to the conventional coupling between orbital angular momentum and spin angular momentum.^[^ ^63^ ^]^ Therefore, we hypothesize that the coupling between the CEF1 and the E_g_ mode is not the same as the conventional coupling between orbital and spin angular momenta. There is a coupling coefficient before the derived Δ*J*_ω_ (see Equation (S1), Supporting Information), leading to non‐integer Δ*J*_ω_ unless it is zero to begin with. As a consequence, only $|\psi_{0}^{+}\rangle \to |\psi_{1}^{+}\rangle ⊗|E_{\text{g,+}}\rangle$, $|\psi_{0}^{-}\rangle \to |\psi_{1}^{-}\rangle ⊗|E_{\text{g,}-}\rangle$, $|\psi_{0}^{+}\rangle \to |\psi_{1}^{-}\rangle ⊗|E_{\text{g,+}}\rangle$, and $|\psi_{0}^{-}\rangle \to |\psi_{1}^{+}\rangle ⊗|E_{\text{g,}-}\rangle$ that have zero angular momentum can satisfy the angular momentum conservation rule in the co‐circular polarization configuration, and other transitions are forbidden in any polarization channel. Moreover, as discussed previously, since $|\psi_{0}^{+}\rangle \to |\psi_{1}^{-}\rangle$ and $|\psi_{0}^{-}\rangle \to |\psi_{1}^{+}\rangle$ are of a higher order and much less probable, only $|\psi_{0}^{+}\rangle \to |\psi_{1}^{+}\rangle ⊗|E_{\text{g,+}}\rangle$ and $|\psi_{0}^{-}\rangle \to |\psi_{1}^{-}\rangle ⊗|E_{\text{g,}-}\rangle$ have detectable signals (see Figure 4d). This could explain why other transitions and the other VBS modes are not observed.

## 3 Conclusion

We have used polarized field‐dependent Raman spectroscopy to elucidate a vibronic bound state in the quantum spin liquid, NaYbSe_2_. Of course, future direct infrared excitation of the modes explored in this manuscript could prove to be a more efficient method for direct control of angular momentum transfer. We have measured quantized angular momentum transfer Δ*J*_*z*_ = ±ℏ between an orbital and a phonon subsystem due to strong coupling between orbitals and *E*_g_ phonons. This was verified by observing clean selection rules between the ground and excited‐state orbital levels as well as the vibronic bound state. This transfer of angular momentum may enable the on‐demand creation of phonon condensates carrying macroscopic angular momentum, though the quantum many‐body physics of the orbital‐phonon interactions does require further study. If the phonon band structure is modified by this coupling, it may also be possible to access the resulting Berry curvature and probe the robustness of potential topological invariants. Additionally, the observation of photon‐ and CEF‐mediated angular‐momentum transfer suggests that there may be many more possible routes to the creation and control of phononic angular momentum by coupling to electronic, spin, and orbital degrees of freedom in a given system. This finding thus creates a framework that may ultimately enable the transduction of possible QSL ground states to experimentally accessible VBSs.

## 4 Experimental Section

### Sample Details

Single crystals of NaYbSe_2_ were synthesized from NaCl (crystalline powder, 99+%), Yb (ingot, 99.9%), and Se (powder, 99.999%) via the flux method. The finely ground flux mixtures of NaCl, Yb, and Se with a molar ratio of 20:1:2.4 were heated slowly to 850 °C in a vacuum. After 2 weeks, the furnace was cooled to room temperature at a rate of 40 °C h^−1^. The laboratory‐grown single crystals of NaYbSe_2_ were separated from excess alkali halide flux by washing with deionized water and isopropyl alcohol inside a fume‐hood. CsYbSe_2_ single crystals were grown using a related flux method that is described in more detail in previous work.^[^ ^34^ ^]^

### Raman Spectroscopy

Variable temperature Raman spectra were acquired in a Montana Instruments closed‐cycle cryostat using an in‐vacuum objective with a numerical aperture of 0.85. A 1.5 mW, 532.03 nm continuous wave laser excited the sample in an out‐of‐plane back scattering geometry (beam path ‖ **c**). Rayleigh scattering was minimized with either a set of three Optigrate volume Bragg gratings or a set of Semrock RazorEdge ultrasteep dichroic and long‐pass edge filters with cutoff at 90 cm^−1^, and spectra were acquired with a 30 s exposure time.

Magnetic‐field‐dependent Raman spectra were acquired at a fixed temperature of *T* = 4 K for **H** ‖ **c** in a customized Leiden dilution refrigerator with free space optical access to the sample at the mixing chamber stage.^[^ ^64^ ^]^ The spectra were taken with an Andor Kymera 193 spectrograph (2400 line mm^−1^ grating) and a Newton EMCCD DU970P‐BV camera. The same laser and filters were used as with the variable temperature measurements, with the laser power set to 1.0 mW and a typical exposure time of 300 s per spectrum. Achromatic half‐wave plates and quarter‐wave plates were mounted on rotators for automated polarization control in both microscopes.

### Crystal Field Hamiltonian

For a review of the approaches used here, see, for example, Baqrtolomé et al.^[^ ^65^ ^]^ The CEF Hamiltonian for NaYbSe_2_ within the point charge approximation^[^ ^23^, ^25^, ^32^, ^66^ ^]^ that described the ground state manifold *J* = 7/2 is

(1) $$\begin{array}{cc} H_{\text{CEF}} & =B_{2}^{0}O_{2}^{0}+B_{4}^{0}O_{4}^{0}+B_{4}^{3}O_{4}^{3}\\ & \;+B_{6}^{0}O_{6}^{0}+B_{6}^{3}O_{6}^{3}+B_{6}^{6}O_{6}^{6} \end{array}$$

The expected helicity dependence of the CEF excitations follows from the selection rule bridging the two relevant states. The eigenstates for the energy levels described in Equation (1) are:

(2) $$\begin{array}{cc} |\psi_{\text{0, 1, 2, 3}}^{+}\rangle & =\sum_{m_{j}=-\frac{7}{2},-\frac{5}{2}\text{…}\frac{5}{2},\frac{7}{2}}c_{m_{j}}^{+}\left|\frac{7}{2},m_{j}\right\rangle \\ |\psi_{\text{0, 1, 2, 3}}^{-}\rangle & =\sum_{m_{j}=-\frac{7}{2},-\frac{5}{2}\text{…}\frac{5}{2},\frac{7}{2}}c_{m_{j}}^{-}\left|\frac{7}{2},m_{j}\right\rangle \end{array}$$

where the relative weights $c_{m_{j}}^{\pm }$ are determined by $B_{2}^{0}$, $B_{4}^{0}$, $B_{4}^{3}$, $B_{6}^{0}$, $B_{6}^{3}$, and $B_{6}^{6}$. The eigenstates are shown in Figure 1b. Due to the threefold symmetry of the Yb^3 +^ environment, a threefold periodicity and hence angular momentum folding, analogous to the the Umklapp process for linear momentum^[^ ^62^ ^]^ was expected. Without further constraints, the six parameters have enough degrees of freedom to fit experimentally observed energy levels. More than one set of CEF parameters that minimize the error might exist and the order of the eigenstates might not be the same across those sets. For example, the little group spanned by the special pair with single angular momentum eigenstate $|\frac{7}{2},\frac{3}{2}\rangle$ or $|\frac{7}{2},-\frac{3}{2}\rangle$ was assigned to CEF1 in Zhang et al.^[^ ^23^ ^]^ and CEF2 in Scheie et al.^[^ ^37^ ^]^ Schimidt et al. pointed out that they cannot be the ground state due to observed in‐plane field dependence at low temperatures.^[^ ^25^ ^]^ In addition to the Zeeman dependence *H*_**B**_ = −*g*_*J*_μ_*B*_ **J**_*z*_ · **B**, several additional corrections have been considered in the literature. For example, Pocs et al.^[^ ^32^ ^]^ considered an *H*_XXZ_ term. Zhang et al. considered anisotropic spin–spin interactions.^[^ ^67^ ^]^

In the context of magnetostrictive coupling with CEF modes, Callan et al. considered an additional term $H_{\text{me}}=-\sum_{\Gamma_{\nu }}\zeta \left(\Gamma_{\nu }\right)\;u\left(\Gamma_{\nu }\right)\;Q\left(\Gamma_{\nu }\right)$ where ζ is the coupling strength, *u*(Γ_ν_) are phonon operators, and *Q*(Γ_ν_) is the transformed phonon mode octupolar operator on the CEF manifold. The Callen–Callen^[^ ^68^, ^69^ ^]^ magnetoelastic interaction is quadruplar. The quadruple operator (*l* = 2) is given by *A*_1g_ + 2*E*_g_. Hence,

(3) $$\begin{array}{cc} H_{\text{me}} & =-\sum_{\Gamma_{\nu }}\zeta \left(\Gamma_{\nu }\right)\;u\left(\Gamma_{\nu }\right)\;Q\left(\Gamma_{\nu }\right)\\ Q(A_{\text{1g}}) & =3J_{z}^{2}-J^{2}\\ Q(E_{g}^{I}) & =\alpha_{1}\left(J_{z}J_{x}+J_{x}J_{z}\right)+\alpha_{2}\left(J_{y}J_{z}+J_{z}J_{y}\right)\\ Q(E_{g}^{\text{II}}) & =\alpha_{3}\left(J_{x}^{2}-J_{y}^{2}\right)+\alpha_{4}\left(J_{x}J_{y}+J_{y}J_{x}\right) \end{array}$$

For $Q(E_{g}^{\text{II}})$, since $J_{x}^{2}-J_{y}^{2}=\left(J_{+}J_{+}+J_{-}J_{-}\right)/2$, it induced Δ**J**_*z*_ = ±2ℏ, while for *J*_*x*_ *J*_*y*_ + *J*_*y*_ *J*_*x*_ = *iJ*_*z*_, Δ**J**_*z*_ = 0. For $Q(E_{g}^{I})$, *J*_*z*_ *J*_*x*_ + *J*_*x*_ *J*_*z*_ and *J*_*z*_ *J*_*x*_ + *J*_*x*_ *J*_*z*_ both induces Δ**J**_*z*_ = ±1ℏ.

### Fitting Experimental Helicitiy‐Resolved Data to a CEF Hamiltonian

To fit the experimental data, a general non‐linear model normalization procedure in Mathematica was used. To start, a general set of eigenvalues and eigenvectors as a function of the CEF parameters was obtained by direct diagonalization of the CEF Hamiltonian. The eigenvectors were sorted based on their eigenvalues for each test parameter space. Then the cost function was defined by the sum of the squared errors between transitions and the data, with both inter‐branch and intra‐branch transitions considered. The selection rules were added as a term in the cost function when necessary. The final CEF parameters were the set that minimized the cost function. However, it was noted that the field dependence of the model was only qualitatively accurate not quantitatively: the experimentally observed levels were only 30% to 60% of the level shift in magnetic field. It was reported that there was non‐negligible magnetoelastic coupling and the field dependence needed to include corresponding terms in the Hamiltonian.

### Bayesian Inference

Bayesian inference was employed to extract spectral parameters such as peak positions and widths. This approach is based on Bayes' rule: $P\left(\theta |y\right)=\frac{P(y|\theta )P(\theta )}{P(y)}$. In the context of spectral data, the priors *P*(θ) were the *true* distribution of the parameters such as peak position, peak height, and peak width. The likelihood *P*(*y*|θ) was the experimental data. The posterior *P*(θ|*y*) was the conditional distribution of the experiment parameters given the experimental data. *P*(*y*) was a normalization factor. The distribution of the priors of the parameters was assumed to be Gaussian or uniform. Additional noise, offset, and slope were added to capture backgrounds unrelated to the peak parameters. To carry out the inference, the Hamiltonian Monte Carlo Python package PyMC3 ^[^ ^54^ ^]^ was used. A hierarchical model was constructed to concurrently extract peak parameters from a family of spectra, such as the temperature dependence or magnetic field dependence. A no U‐Turns (NUTS) sampler was used with four chains with 3000 samples per chain. It takes between 20 min to 3 h for a peak over a dataset to converge.

## Conflict of Interest

The authors declare no conflict of interest.

## Author Contributions

All authors discussed the results thoroughly. Y.‐Y.P., C.E.M., and B.J.L. performed magneto‐Raman measurements. L.L. performed Raman tensor analysis. G.P. and J.X. grew the samples. Y.‐Y.P and L.L. did the data analysis with inputs from L.L., M.C., J.S.G., and B.J.L. X.L. and L.L. performed DFT calculations. A.S.S., D.P., S.W., and B.J.L initiated and oversaw the project. Y.‐Y.P., L.L. and B.J.L wrote most of the manuscript with contributions from all authors.

## Supporting information

Supporting Information

## Data Availability

The data that support the findings of this study are available from the corresponding author upon reasonable request.
