# Supplementary material for: Angular‐Momentum Transfer Mediated by a Vibronic‐Bound‐State
Source: Adv Sci (Weinh). 2023 Nov 9;11(2):2304698. doi: 10.1002/advs.202304698 (PMC10787099; doi:10.1002/advs.202304698)
Supplement: Supplementary file 1 — Supporting Information [file ADVS-11-2304698-s001.pdf]

ADVANCED SCIENCE
Open Access

Supporting Information

for *Adv. Sci.*, DOI 10.1002/advs.202304698

Angular-Momentum Transfer Mediated by a Vibronic-Bound-State

Yun-Yi Pai, Claire E. Marvinney, Ganesh Pokharel, Jie Xing, Haoxiang Li, Xun Li, Michael Chilcote, Matthew Brahlek, Lucas Lindsay, Hu Miao, Athena S. Sefat, David Parker, Stephen D. Wilson, Jason S. Gardner, Liangbo Liang and Benjamin J. Lawrie**

Supplementary Information for Angular-Momentum Transfer Mediated by a Vibronic-Bound-State

Yun-Yi Pai,[1,2,*] Claire E. Marvinney,[1,2] Ganesh Pokharel,[3] Jie Xing,[1] Haoxiang Li,[1] Xun Li,[1] Michael Chilcote,[1,2] Matthew Brahlek,[1,2] Lucas Lindsay,[1] Hu Miao,[1] Athena S. Sefat,[1] David Parker,[1] Stephen D. Wilson,[3] Jason S. Gardner,[1] Liangbo Liang,[4,†] and Benjamin J. Lawrie[1,2,‡]

[1]*Materials Science and Technology Division, Oak Ridge National Laboratory, Oak Ridge, TN 37831, USA*

[2]*Quantum Science Center, Oak Ridge, Tennessee 37831, USA*

[3]*Materials Department and California Nanosystems Institute, University of California Santa Barbara, Santa Barbara, CA 93106, USA*

[4]*Center for Nanophase Materials Sciences, Oak Ridge National Laboratory, Oak Ridge, TN 37831, USA*

(Dated: September 23, 2023)

* yunyipai@gmail.com

† liangl1@ornl.gov

‡ lawriebj@ornl.gov

CONTENTS

I. Phonon calculation details 2

II. Temperature Dependence for Higher Energy Band 4

III. Temperature Dependence for XX and XY polarization 8

IV. Full Magneto-Raman for both $NaYbSe_2$ and $CsYbSe_2$ at $T = 4$K 9

V. Analysis of transitions for the VBS mode 12

References 13

I. PHONON CALCULATION DETAILS

Density functional theory (DFT) calculations were performed based on the projector augmented wave method (PAW) as implemented in the Vienna Ab-initio Simulation Package (VASP) [1–4] to obtain the phonon dispersion relationship in $NaYbSe_2$, . The generalized gradient approximation, parameterized by Perdew, Burke, and Ernzerhof (PBE) [5] was used for exchange-correlations. A 520 eV kinetic energy cutoff in the plane-wave expansion and energy convergence criteria of 10^{-6} eV were employed. Ionic relaxations were performed until Hellmann-Feynman forces converged to 10^{-4} meV/Å. The structure was relaxed with a Γ-centered 15×15×3 k-mesh. The harmonic interatomic force constants (IFCs) were calculated using the finite displacement method implemented in the phonopy package [6] in a 3×3×1 supercell with Γ-centered 5×5×3 k-meshes. The DFT+U method [7] was used to include the Coulomb correlations with U_{eff}=6 eV [7] for Yb atoms. Van der Waals interactions were taken into account via DFT-D3 method [8].

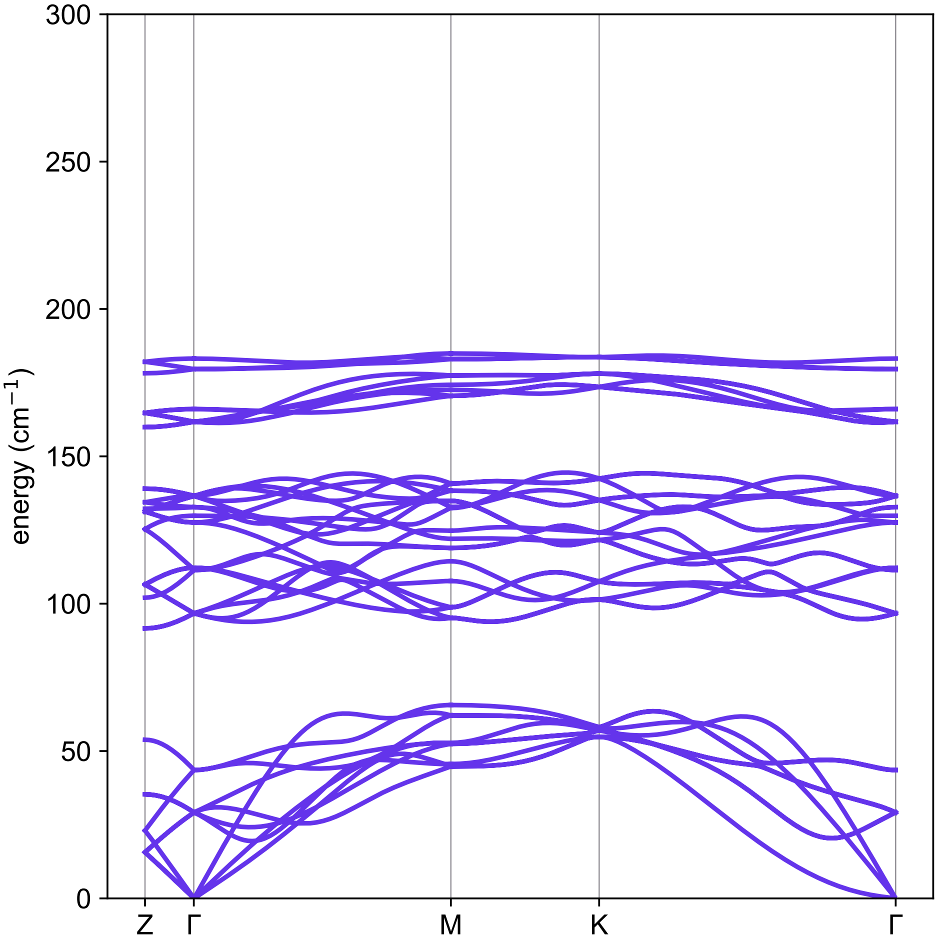

FIG. S1. Calculated phonon dispersion.

II. TEMPERATURE DEPENDENCE FOR HIGHER ENERGY BAND

Figure S2 (raw spectra) and Figure S3 (contour) shows temperature-dependent unpolarized Raman spectra taken from $T = 3.3$ K to $T = 270$ K. The spectra were taken with Semrock dichroic and longpass filters with cutoff at 90 cm^{-1} instead of a set of volume Bragg gratings. Similar to $CsYbSe_2$ [9], a multitude of possible combination modes (e.g., possible CEF1 + CEF2) – behavior of resonant Raman excitation – is present.

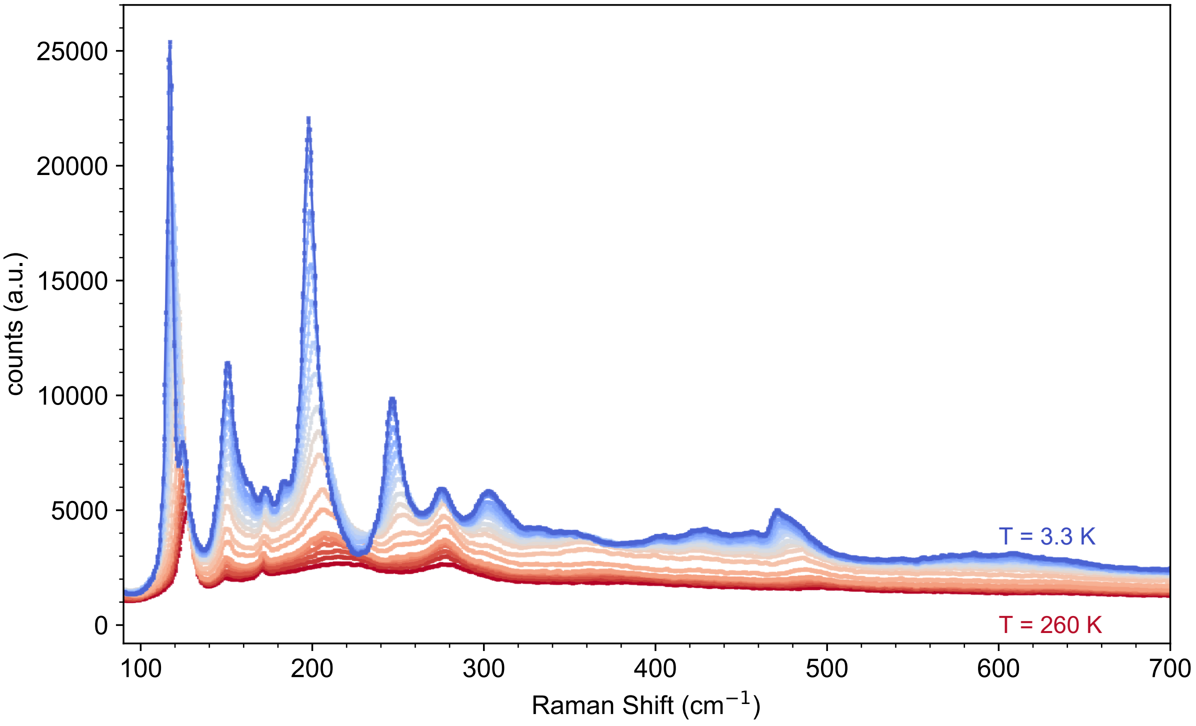

FIG. S2. Raman spectra as function of temperature from $T = 3.3$ K to $T = 270$ K.

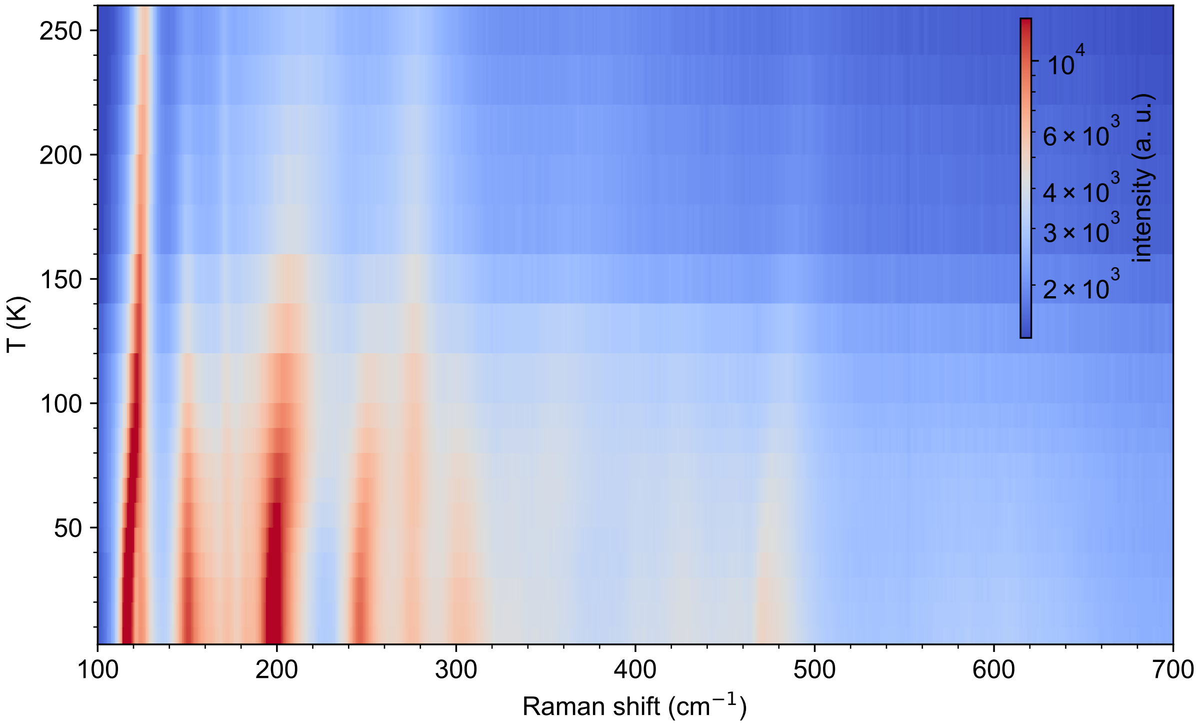

FIG. S3. Contour plot for Raman spectra as function of temperature from T = 3.3 K to T = 270 K. Same data as in Figure S2.

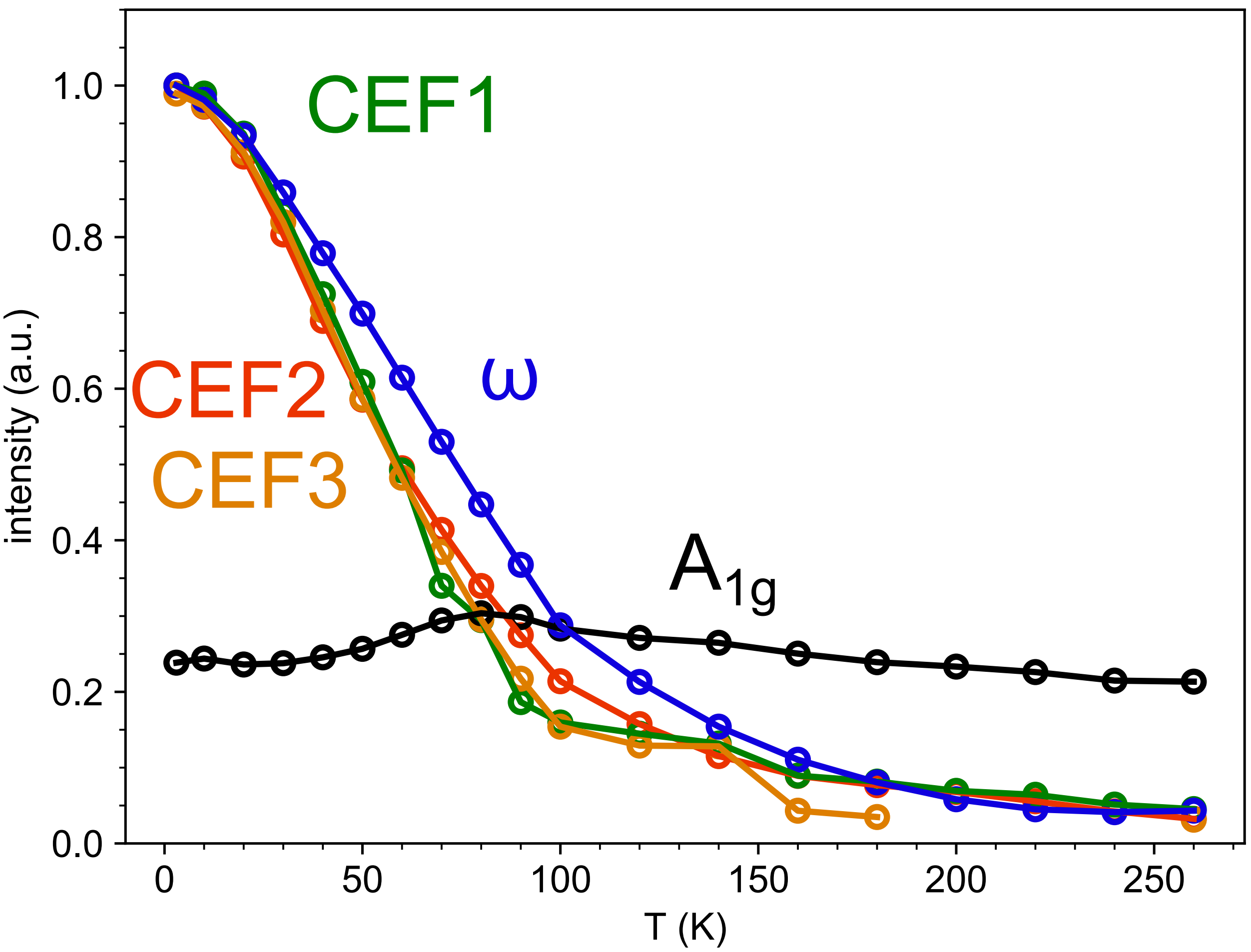

FIG. S4. Extraced peak heights as a function of temperature for CEF1, CEF2, CEF3, ω and A_{1g}.

Figure S5 shows the peak positions extracted from Figure S2 using Bayesian inference [10]. In each subplot, the trace is the median of the posterior distribution of the peak position or peak width, and the two shades of different opacities are corresponding to 1σ (more opaque) and 2σ (more transparent) bands of the posterior distribution.

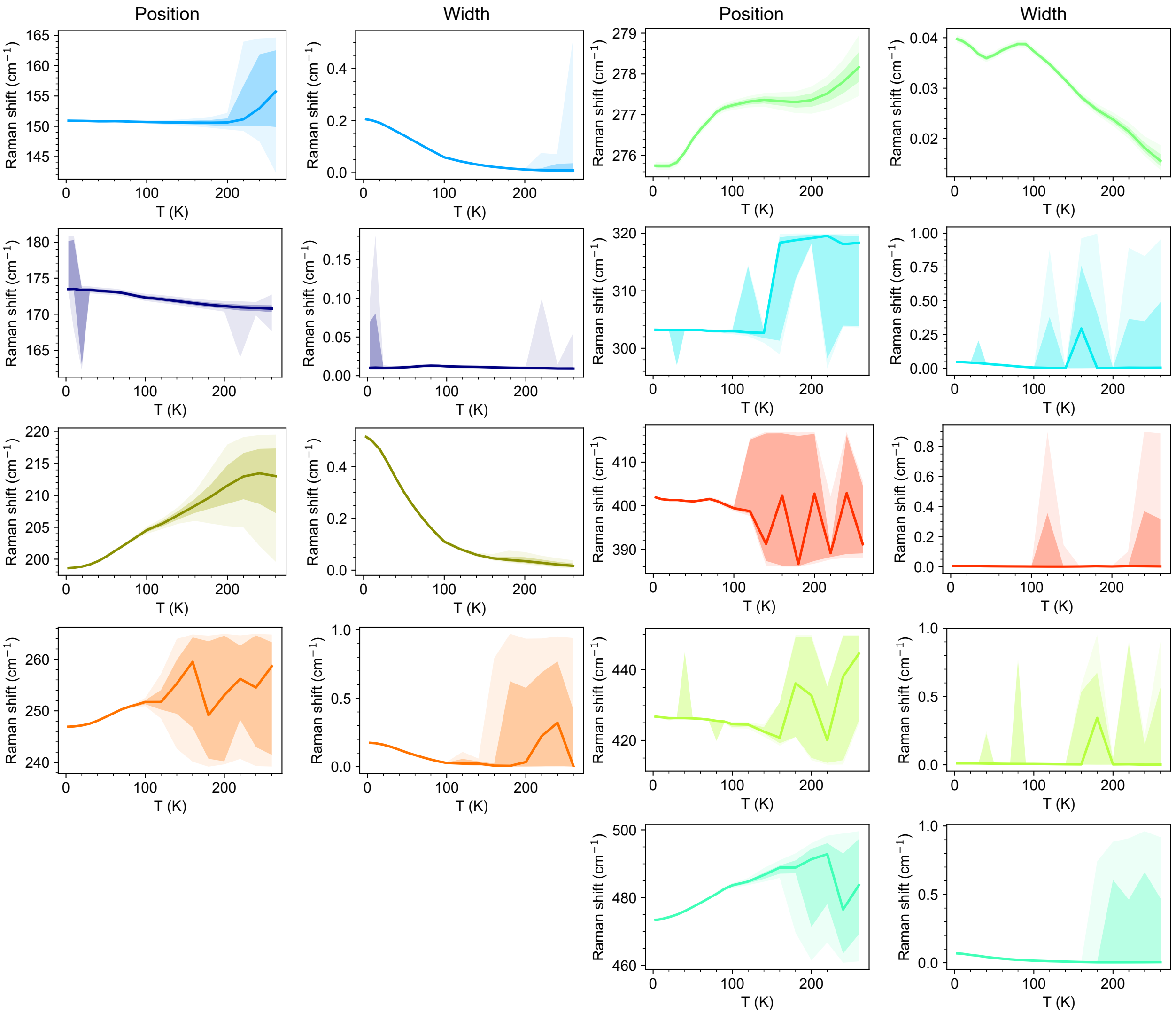

FIG. S5. Peak postitions extracted from Figure S2.

III. TEMPERATURE DEPENDENCE FOR XX AND XY POLARIZATION

Figure S6 shows temperature-dependent Raman spectra taken from $T = 3.3$ K to $T = 270$ K with polarization configuration XX and XY.

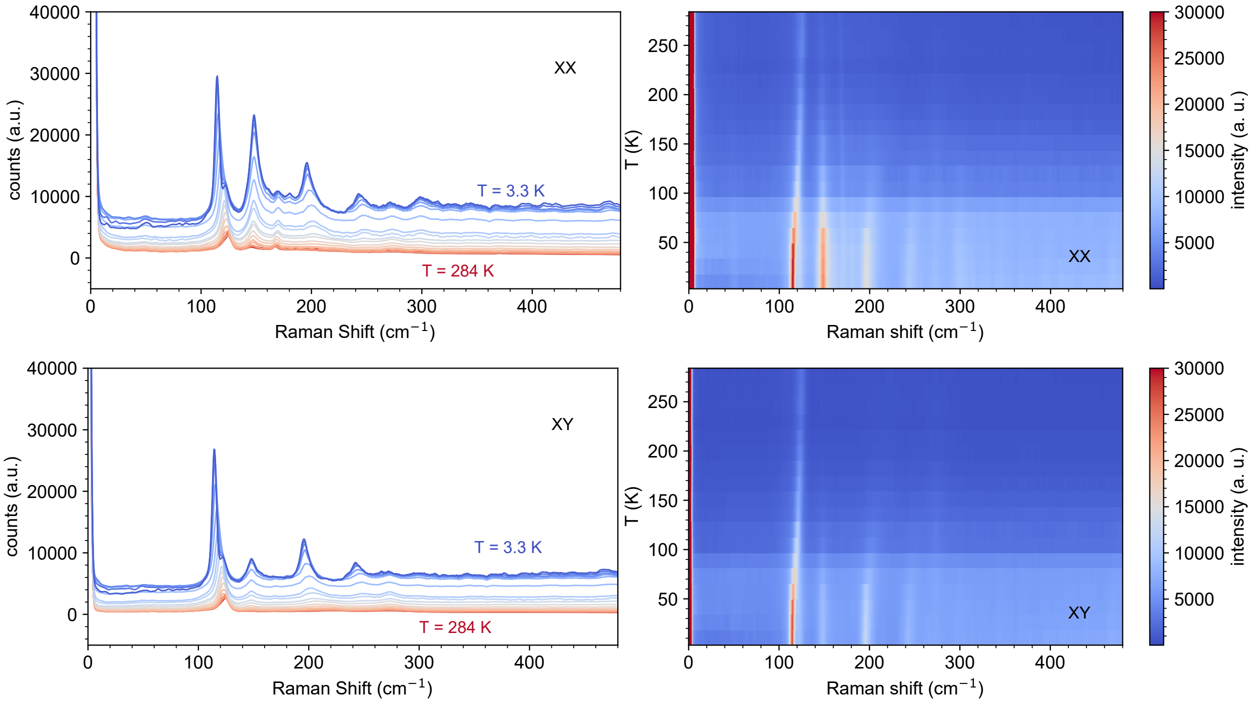

FIG. S6. Raman spectra taken from $T = 3.3$ K to $T = 270$ K with polarization configuration XX and XY.

IV. FULL MAGNETO-RAMAN FOR BOTH $NaYbSe_2$ AND $CsYbSe_2$ AT $T = 4K$

Figure S7 shows the original magneto-Raman spectra for $NaYbSe_2$ and Figure S8 for $CsYbSe_2$. The magnetic field dependence given polarization configuration for the CEF modes are similar for $NaYbSe_2$ and $CsYbSe_2$, i.e., (i) with (σ_{incident}, σ_{scatter}) = (σ^+, σ^-) configuration, the CEF1 and CEF2 shift toward higher energy in positive field, and CEF3 toward lower energy and (ii) the trends are inverted with inverted polarization configurations or inverted field. However, the ω is observed only in a subset of spatial locations [9], and with much weaker intensity, likely due to larger detuning between the E_{2g}^2 and CEF1 in $CsYbSe_2$. Small residual peaks that violate the proposed selection rules are present in both datasets: for instance, a small ω peak is present in Figure S7 (c) and Figure S7 (e) due to less than ideal polarization contrast in the optics train. For $CsYbSe_2$, the extinction ratio of the selection rules are not as high as that of $NaYbSe_2$, suggesting that the selection rules (and hence the assignment of the states) proposed in the text may only apply to $NaYbSe_2$.

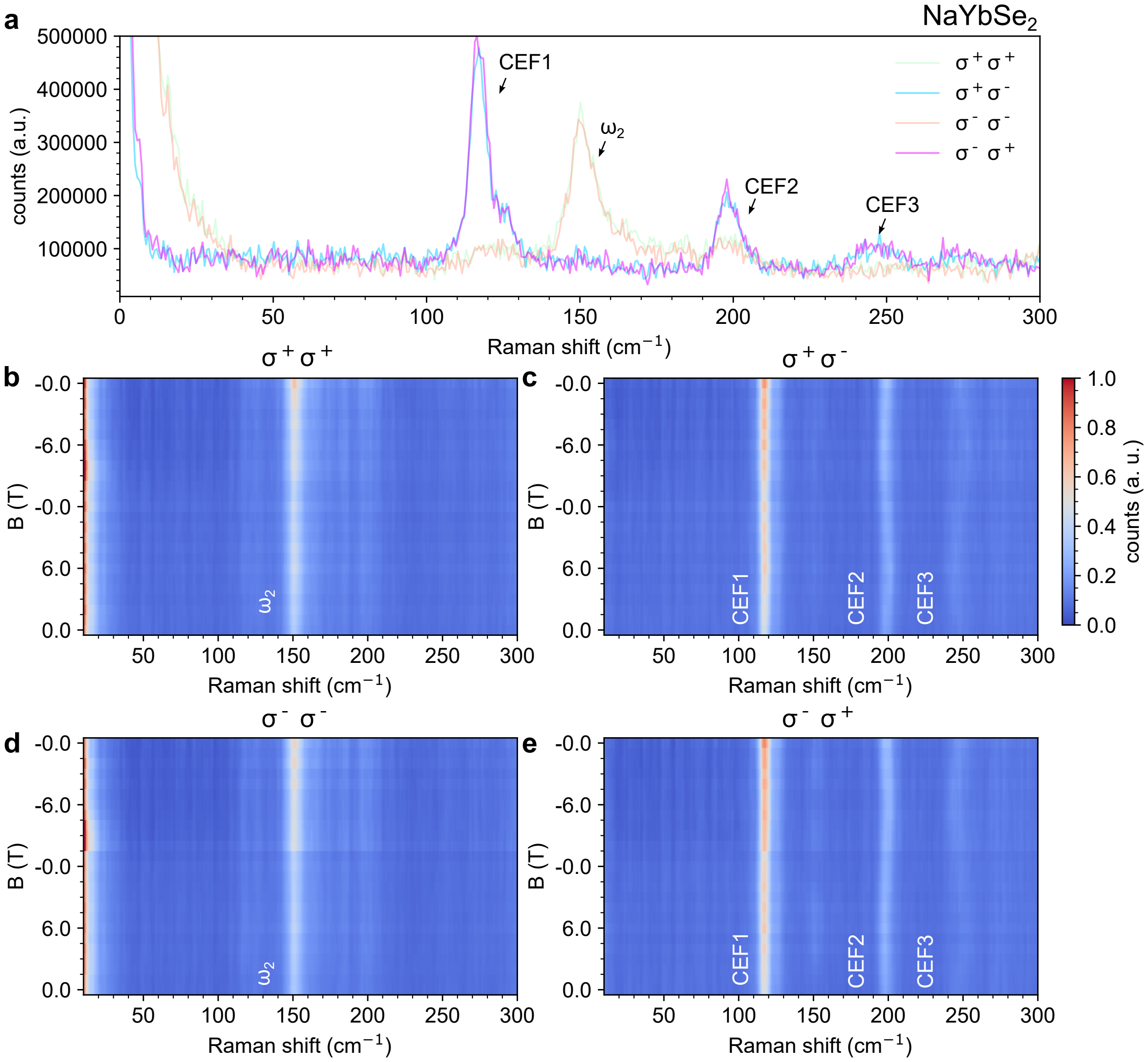

FIG. S7. Helicity-resolved magnetic field dependence of CEFs and ω for $NaYbSe_2$ at $T = 4$ K. (a) at 0T, (b) (σ^+, σ^+). (c) (σ^+, σ^-). (d) (σ^-, σ^-). (e) (σ^-, σ^+).

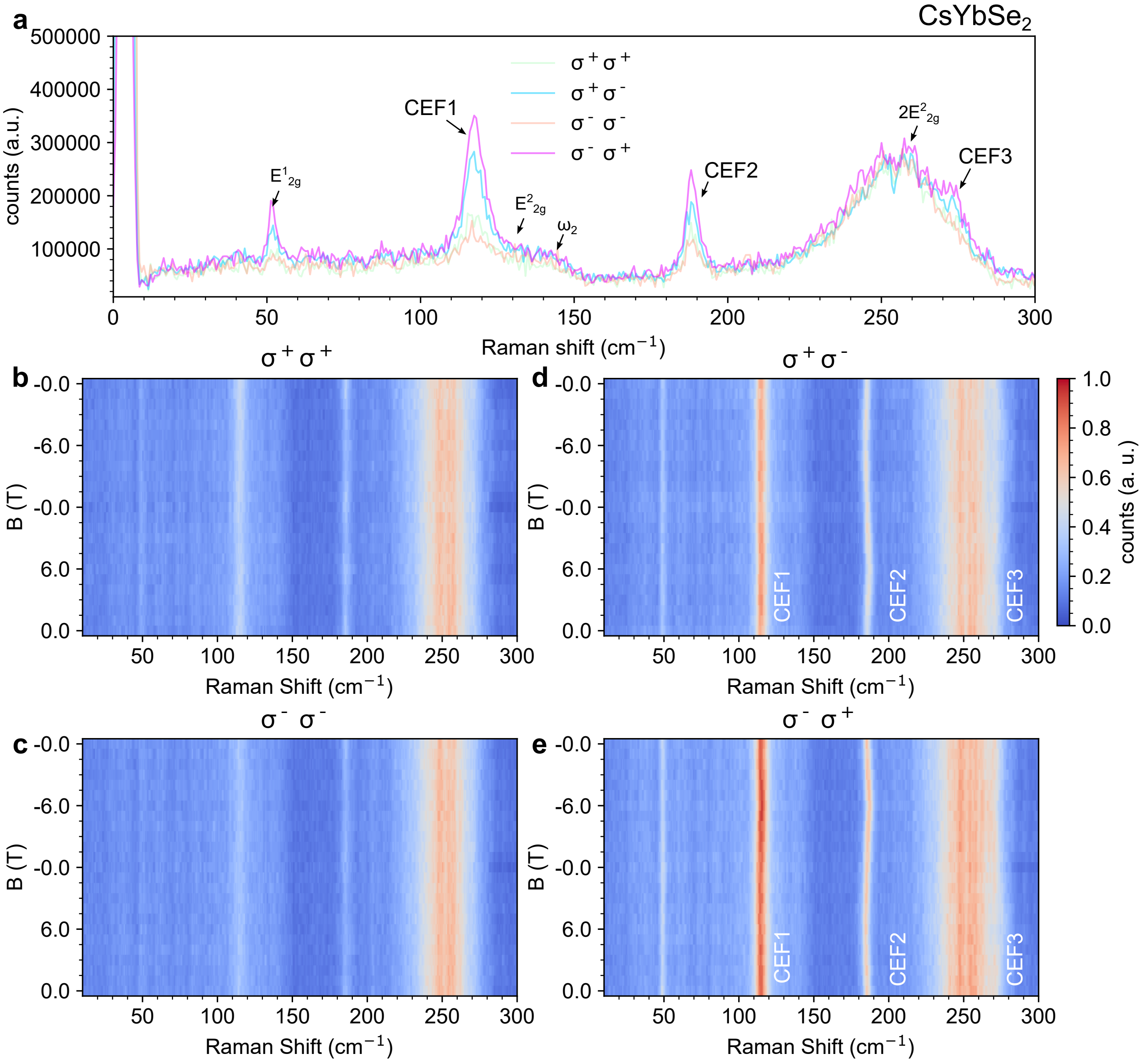

FIG. S8. Helicity-resolved magnetic field dependence of CEFs and ω for $CsYbSe_2$ at $T = 4$ K. (a) at 0T, (b) (σ^+, σ^+). (c) (σ^+, σ^-). (d) (σ^-, σ^-). (e) (σ^-, σ^+).

V. ANALYSIS OF TRANSITIONS FOR THE VBS MODE

The VBS ω is a hybridized state between the CEF1 and the E_g mode. As discussed in the main text, CEF1 can arise from four possible transitions: $|\psi_0^+\rangle \rightarrow |\psi_1^+\rangle$, $|\psi_0^-\rangle \rightarrow |\psi_1^-\rangle$, $|\psi_0^+\rangle \rightarrow |\psi_1^-\rangle$, and $|\psi_0^-\rangle \rightarrow |\psi_1^+\rangle$, while E_g can be $E_{g,+}$ and $E_{g,-}$ depending on the sign of the angular momentum. Therefore, the ω could originate from eight possible transitions: $|\psi_0^+\rangle \rightarrow |\psi_1^+\rangle \otimes |E_{g,+}\rangle$, $|\psi_0^+\rangle \rightarrow |\psi_1^+\rangle \otimes |E_{g,-}\rangle$, $|\psi_0^-\rangle \rightarrow |\psi_1^-\rangle \otimes |E_{g,+}\rangle$, $|\psi_0^-\rangle \rightarrow |\psi_1^-\rangle \otimes |E_{g,-}\rangle$, $|\psi_0^+\rangle \rightarrow |\psi_1^-\rangle \otimes |E_{g,+}\rangle$, $|\psi_0^+\rangle \rightarrow |\psi_1^-\rangle \otimes |E_{g,-}\rangle$, $|\psi_0^-\rangle \rightarrow |\psi_1^+\rangle \otimes |E_{g,+}\rangle$, and $|\psi_0^-\rangle \rightarrow |\psi_1^+\rangle \otimes |E_{g,-}\rangle$. For $|\psi_0^+\rangle \rightarrow |\psi_1^+\rangle \otimes |E_{g,+}\rangle$, the z-component of angular momentum of $|\psi_0^+\rangle \rightarrow |\psi_1^+\rangle$ is $+2\hbar$ or $-\hbar$ while that of $E_{g,+}$ is $+\hbar$, and thus the z-component of the total angular momentum is $+3\hbar$ or 0 ($\Delta J_\omega = +3\hbar$ or 0), as shown in Figures 4a and 4d; in contrast, for $|\psi_0^+\rangle \rightarrow |\psi_1^+\rangle \otimes |E_{g,-}\rangle$, the z-component of angular momentum of $E_{g,-}$ is $-\hbar$, and thus the z-component of the total angular momentum is $+\hbar$ or $-2\hbar$ ($\Delta J_\omega = +\hbar$ or $-2\hbar$). Similarly, we can determine the z-component of angular momentum of the ω for other six possible transitions. It is summarized below:

$$\begin{aligned}
&\text{for } |\psi_0^+\rangle \rightarrow |\psi_1^+\rangle \otimes |E_{g,+}\rangle, \Delta J_\omega = +3\hbar \text{ or } 0; && \text{for } |\psi_0^+\rangle \rightarrow |\psi_1^+\rangle \otimes |E_{g,-}\rangle, \Delta J_\omega = +\hbar \text{ or } -2\hbar;\\
&\text{for } |\psi_0^-\rangle \rightarrow |\psi_1^-\rangle \otimes |E_{g,+}\rangle, \Delta J_\omega = -\hbar \text{ or } +2\hbar; && \text{for } |\psi_0^-\rangle \rightarrow |\psi_1^-\rangle \otimes |E_{g,-}\rangle, \Delta J_\omega = -3\hbar \text{ or } 0;\\
&\text{for } |\psi_0^+\rangle \rightarrow |\psi_1^-\rangle \otimes |E_{g,+}\rangle, \Delta J_\omega = 0; && \text{for } |\psi_0^+\rangle \rightarrow |\psi_1^-\rangle \otimes |E_{g,-}\rangle, \Delta J_\omega = -2\hbar;\\
&\text{for } |\psi_0^-\rangle \rightarrow |\psi_1^+\rangle \otimes |E_{g,+}\rangle, \Delta J_\omega = +2\hbar; && \text{for } |\psi_0^-\rangle \rightarrow |\psi_1^+\rangle \otimes |E_{g,-}\rangle, \Delta J_\omega = 0.
\end{aligned} \quad (1)$$

According to the discrete angular momentum conservation rule $|\Delta J_{\text{photon}} + \Delta J_\omega|\hbar \text{modulo} 3 = 0$, the eight transitions are allowed in either cross-circular or co-circular polarization, which is inconsistent with the experimental observation that the ω can only be observed in co-circular polarization. We note that the above discussions have a basic assumption that the total angular momentum between the CEF1 and the E_g mode follows the same rule for the conventional coupling between orbital angular momentum and spin angular momentum. However, the electron-phonon coupling may be more complicated according to a recent work [11]: Coupling $= -\alpha \mathbf{L}^{\text{phonon}} \cdot \langle \mathbf{J}^{\text{electron}} \rangle$, where α is a coupling constant in units of the inverse of the moment of inertia. Based on this concept, we hypothesize that the coupling between the CEF1 and the E_g mode is not the same as the conventional coupling between orbital and spin angular momenta, and there is a coupling constant before the derived ΔJ_ω

above in Eq. 1, leading to non-integer ΔJ_ω unless it is zero to begin with. Therefore, only $|\psi_0^+\rangle \to |\psi_1^+\rangle \otimes |\mathrm{E}_{\mathrm{g},+}\rangle$, $|\psi_0^-\rangle \to |\psi_1^-\rangle \otimes |\mathrm{E}_{\mathrm{g},-}\rangle$, $|\psi_0^+\rangle \to |\psi_1^-\rangle \otimes |\mathrm{E}_{\mathrm{g},+}\rangle$, and $|\psi_0^-\rangle \to |\psi_1^+\rangle \otimes |\mathrm{E}_{\mathrm{g},-}\rangle$ that have zero angular momentum can satisfy the angular momentum conservation rule in the co-circular polarization. This can explain why the ω can only be experimentally observed in co-circular polarization. Furthermore, as discussed in the main text, since $|\psi_0^+\rangle \to |\psi_1^-\rangle$ and $|\psi_0^-\rangle \to |\psi_1^+\rangle$ are of a higher order and less probable, we assign the vibronic bound state to $|\psi_0^+\rangle \to |\psi_1^+\rangle \otimes |\mathrm{E}_{\mathrm{g},+}\rangle$ and $|\psi_0^-\rangle \to |\psi_1^-\rangle \otimes |\mathrm{E}_{\mathrm{g},-}\rangle$ (see Figure 4d).

---

[1] Blöchl, P. E. Projector augmented-wave method. *Phys. Rev. B* **50**, 17953–17979 (1994). URL `https://link.aps.org/doi/10.1103/PhysRevB.50.17953`.

[2] Kresse, G. & Furthmller, J. Efficiency of ab-initio total energy calculations for metals and semiconductors using a plane-wave basis set. *Computational Materials Science* **6**, 15–50 (1996). URL `https://www.sciencedirect.com/science/article/pii/0927025696000080`.

[3] Kresse, G. & Furthmüller, J. Efficient iterative schemes for ab initio total-energy calculations using a plane-wave basis set. *Phys. Rev. B* **54**, 11169–11186 (1996). URL `https://link.aps.org/doi/10.1103/PhysRevB.54.11169`.

[4] Kresse, G. & Joubert, D. From ultrasoft pseudopotentials to the projector augmented-wave method. *Phys. Rev. B* **59**, 1758–1775 (1999). URL `https://link.aps.org/doi/10.1103/PhysRevB.59.1758`.

[5] Perdew, J. P., Burke, K. & Ernzerhof, M. Generalized gradient approximation made simple. *Phys. Rev. Lett.* **77**, 3865–3868 (1996). URL `https://link.aps.org/doi/10.1103/PhysRevLett.77.3865`.

[6] Togo, A. & Tanaka, I. First principles phonon calculations in materials science. *Scripta Materialia* **108**, 1–5 (2015).

[7] Dudarev, S. L., Botton, G. A., Savrasov, S. Y., Humphreys, C. J. & Sutton, A. P. Electron-energy-loss spectra and the structural stability of nickel oxide: An lsda+u study. *Phys. Rev. B* **57**, 1505–1509 (1998). URL `https://link.aps.org/doi/10.1103/PhysRevB.57.1505`.

[8] Grimme, S., Antony, J., Ehrlich, S. & Krieg, H. A consistent and accurate ab initio parametrization of density functional dispersion correction (dft-d) for the 94 elements h-pu. *The Journal of Chemical Physics* **132**, 154104 (2010). URL `https://doi.org/10.1063/1.`

3382344. https://doi.org/10.1063/1.3382344.

[9] Pai, Y.-Y. *et al.* Mesoscale interplay between phonons and crystal electric field excitations in quantum spin liquid candidate csybse2. *J. Mater. Chem. C* – (2022). URL http://dx.doi.org/10.1039/D1TC05934C.

[10] Salvatier, J., Wiecki, T. V. & Fonnesbeck, C. Probabilistic programming in python using pymc3. *PeerJ Computer Science* **2**, e55 (2016). URL https://doi.org/10.7717/peerj-cs.55.

[11] Geilhufe, R. M. & Hergert, W. Electron magnetic moment of transient chiral phonons in $ktao_3$. *Phys. Rev. B* **107**, L020406 (2023). URL https://link.aps.org/doi/10.1103/PhysRevB.107.L020406.
